# Post-marketing safety surveillance of voclosporin: an observational, pharmacovigilance study leveraging faers database study on the safety of voclosporin

**DOI:** 10.3389/fphar.2025.1506760

**Published:** 2025-05-19

**Authors:** Zhi Wang, Yanhua Lang, Xuyan Liu, Yuxuan Wang, Leping Shao

**Affiliations:** ^1^ Department of Nephrology, The First Affiliated Hospital of Xiamen University, Xiamen, China; ^2^ Department of Public Health, The First Affiliated Hospital of Xiamen University, Xiamen, China; ^3^ Department of Nephrology, The Affiliated Qingdao Municipal Hospital of Qingdao University, Qingdao, China; ^4^ Department of Emergency, Qingdao Hospital, University of Health and Rehabilitation Sciences (Qingdao Municipal Hospital), Qingdao, China

**Keywords:** voclosporin, lupus nephritis, pharmacovigilance, disproportionality analysis, FAERS database

## Abstract

**Background:**

Voclosporin is a novel calcineurin inhibitor approved for the treatment of adult active lupus. Based on the US Food and Drug Administration Adverse Event Reporting System FDA Adverse Event Reporting System database, this study conducted a comprehensive evaluation of the safety characteristics of voclosporin in clinical use.

**Methods:**

We selected data from the FAERS database from the 2021Q1 to 2024Q2. Disproportionality analysis was employed to detect positive signals at the System Organ Class (SOC) and Preferred Term (PT) levels. Our analysis encompassed the time to onset, and subgroup analyses stratified by gender and age.

**Results:**

There are 3,407,094 reports collected from the FAERS database, of which 4,013 reports listed voclosporin as the “primary suspected” drug. Eight positive SOC signals and 138 positive PT signals were identified, including some unexpected signals that were not listed on the drug label such as menstrual disorder (n = 7, ROR = 5.82) and acute pulmonary oedema (n = 3, ROR = 4.16). Gender differences and age differences existed in PT signals related to voclosporin. Nearly one-third of the adverse events occurred within the first month after voclosporin initiation, with a median onset time of 99 days.

**Conclusion:**

This study clarified the characteristics of voclosporin-related adverse drug reactions, providing strong support for clinical monitoring and risk identification of voclosporin.

## Introduction

Lupus nephritis (LN) is the most common and severe complication of systemic lupus erythematosus (SLE), occurring in more than half of SLE patients (Zhou et al., 2018). The pathogenesis of LN includes aberrant activation of immune responses, autoantibody production, and immune mediated renal injury (Obrişcă et al., 2021a). Typically, LN occurs within the first 5 years of the onset of SLE, presenting from asymptomatic proteinuria or hematuria to clinically apparent nephrotic syndrome. Up to 20% of patients eventually progressed to end-stage renal disease (Anders et al., 2020). Steroids, cyclophosphamide, azathioprine, and mycophenolate mofetil remain the current first-line treatment for LN (Lech and Anders, 2013). These non-selective immunosuppressants have significantly improved patient and renal survival in LN (Moroni et al., 2018; Croca et al., 2011). However, there remains a substantial treatment-related morbidity and mortality, which has prompted the development of new therapeutic approaches targeting specific leukocyte subpopulations, such as anti-CD20 antibodies, proteasome inhibitors, and calcineurin inhibitors (CNIs) (Obrişcă et al., 2021b; Yung et al., 2020).

Voclosporin, a novel CNI, has been developed for the treatment of LN. The structure of voclosporin is similar to cyclosporine (CsA), containing a single amino acid substitution. Compared to other CNI, voclosporin exhibited enhanced efficacy, metabolic stability, and safety (Kale et al., 2023). The rationale for treating LN with voclosporin involved preventing excessive immune responses by inhibiting the activation of T cells. Additionally, voclosporin stabilizes the podocyte cytoskeleton, reducing proteinuria and attenuating renal damage in lupus patients (Rafael-Vidal et al., 2021). In the AURA-LV and AURORA 1 randomized controlled trials, voclosporin demonstrated superiority in achieving a complete renal response compared to standard care and received FDA approval in January 2021 as the first oral therapy for LN. Despite improved tolerability over traditional CNIs, voclosporin-related adverse drug reactions (ADRs) were still non negligible, including decreased estimated glomerular filtration rate (eGFR), hypertension, and headaches (Rovin et al., 2019; Rovin et al., 2021). Moreover, voclosporin has a black box warning for potential malignancies and serious infections risk, which may be fatal. Therefore, there is a crucial need to utilize real-world data to identify voclosporin safety signals (Lupkynis, 2021).

## Materials and methods

### Data sources and processing

The data for this study was downloaded from the FAERS database (Bihan et al., 2020), which serves as the primary system for post-marketing surveillance of adverse drug reactions in the United States and is a key avenue for current pharmacovigilance research. “Voclosporin” and its trade name “LUPKYNIS” were used as keywords for drug retrieval. In the FAERS database, drug-event relationships are classified into four categories: primary suspect, secondary suspect, concomitant, and interacting. To enhance the reliability of the analysis, only reports in which voclosporin was designated as the primary suspect (PS) were retained. Ultimately, we extracted adverse drug reactions related to voclosporin spanning from 2021Q1 to 2024Q2. Following FDA suggestions, we selected the most recent FDA_DT with the same PRIMARYID, and when FDA_DT and CASEID were identical, we chose the higher PRIMARYID to eliminate duplicates prior to data analysis. The collected data included demographic information, drugs, therapies, indications, reactions, and outcomes from the adverse reaction reports. We employed the “Medical Dictionary for Regulatory Activities” (MedDRA) to standardize adverse events terms in the Preferred Term (PT) format, subsequently classifying PTs by their respective System Organ Classes (SOCs).

### Statistical analysis

We employed reporting odds ratio (ROR) to investigate the associations between voclosporin and adverse events (AEs). The calculation of the ROR algorithm was based on a 2 × 2 columnar table, with the detailed algorithm presented in Supplementary Table S1. If the lower limit of 95% CI exceeded 1.0 with at least three AE reports, the ROR is a positive signal. Higher ROR values indicated stronger signal intensity, signifying a stronger association between the target drug and the specific AE.

### Subgroup analysis

To identify gender-specific and age-specific risks of ADRs, the ROR was utilized to detect disproportional signals among different subgroups following voclosporin administration, as described previously (Zou et al., 2024). The stratification was specifically into males and females, as well as adults aged 18–64 and the elderly aged 65 and above.

### Time to onset analysis

The time to onset of ADEs caused by voclosporin were defined as the interval between the reported ADEs onset date and the start date of voclosporin administration. This study initially assessed the overall time to ADRs following voclosporin use. Subsequently, based on the results of the disproportionality analysis, the onset times of SOC levels and PT levels were assessed. We summarized the onset times of AEs using the median, interquartile range (IQR), and applied the Kruskal-Wallis test to evaluate whether there were significant differences in the onset times between different SOCs or different PTs.

The statistical significance was set at p < 0.05 for all analyses. All statistical analyses were performed using GraphPad Prism 10 (GraphPad Software, CA, USA) and R 4.3.3.

## Result

### General characteristics

From 2021Q1 to 2024Q2, a total of 3,407,094 case reports were obtained from the FAERS database. There were 4,013 case reports associated with voclosporin as the PS. Of these, AE reports from female accounted for 84.0%, significantly higher than those from males at 14.8%. According to the available age data, the most reports (26.4%) were in the age group of 18–44 years followed closely by 44–64 years (10.8%). The principal source of reports was the United States, contributing 4,006 reports (99.8%), and were generally reported by consumers (n = 3,329, 83.00%). Among the reported outcomes, hospitalization (n = 379, 9.4%) was the most common severe outcome (Table 1).

**TABLE 1:** Clinical characteristics of case reports with voclosporin from the FAERS database.

Characteristics	Case numbers	Case proportion
Gender		
Female	3,369	83.95%
Male	592	14.75%
Unknown	52	1.30%
Age		
<18	14	0.35%
18–44	1,061	26.44%
45–64	434	10.81%
>64	77	1.92%
Unknown	2,427	60.48%
Outcome		
Hospitalization	378	9.42%
Death	24	0.60%
Disability	1	0.02%
Other outcome	371	9.24%
Unknown	3,239	80.71%
Reported person		
Consumer	3,329	82.96%
Physician	279	6.95%
Pharmacist	19	0.47%
Other health-professional	386	9.62%
Reported countries		
United States	4,006	99.83%
Sweden	2	0.05%
United Kingdom	1	0.02%
Spain	4	0.10%

### System organ class signals

**FIGURE 1:**
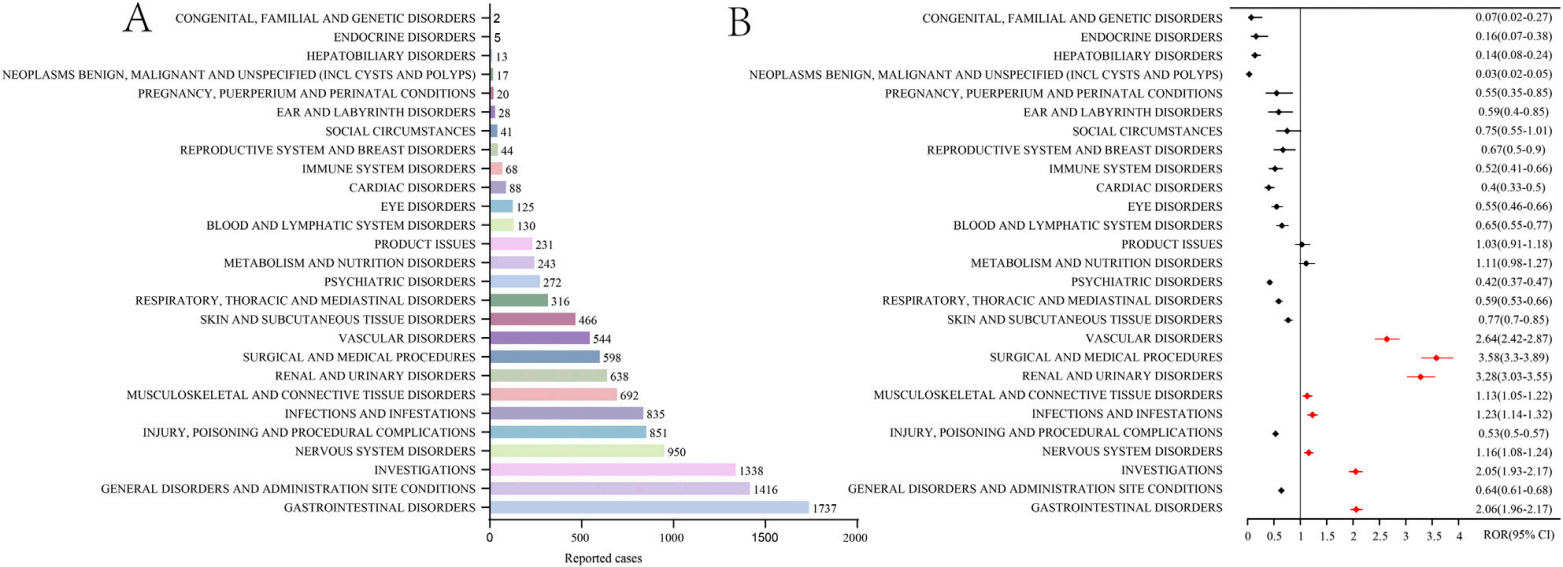
Signals detection at the SOC level. **(A)** The number of adverse events at the SOC level. **(B)** The results of disproportionality analysis based on the ROR algorithm at the SOC level. TheROR values and their 95% confidence intervals (95% CI) are visualized. SOCs with positive signals identified by the ROR algorithm are highlighted in red for clarity.

As shown in Figure 1, the AEs resulted from voclosporin usage impacted 27 SOCs. Among these, eight noteworthy SOCs emerged, meeting ROR algorithms, including gastrointestinal disorders (n = 1737, ROR = 2.03), investigations (n = 1,338, ROR = 2.07), nervous system disorders (n = 950, ROR = 1.12), infections and infestations (n = 835, ROR = 1.27), musculoskeletal and connective tissue disorders (n = 692, ROR = 1.15), renal and urinary disorders (n = 638, ROR = 2.86), surgical and medical procedures (n = 598, ROR = 3.66), vascular disorders (n = 544, ROR = 2.57).

### Preferred terms signals

The AEs associated with voclosporin encompassed 138 PT signals (Supplementary Table S2). To more accurately assess AEs directly related to voclosporin use, this study excluded AEs that could be attributed to drug characteristics or non-pharmacological factors, such as “injury, poisoning and procedural complications” and “product issues.” Tables 2, 3 list the top 50 PTs based on occurrence frequency and ROR values. Common AEs included hypertension (n = 442, ROR = 12.05), headache (n = 404, ROR = 3.79), nausea (n = 342, ROR = 2.62), diarrhea (n = 297, ROR = 2.4), and proteinuria (n = 231, ROR = 62.91), while AEs with higher ROR values primarily involved abnormalities in various investigations results. Most AEs were consistent with the results of clinical trials and the drug instruction. Notably, several unexpected AEs were also identified, including breath odour (n = 4, ROR = 12.5), menstrual disorder (n = 7, ROR = 5.82), acute pulmonary oedema (n = 3, ROR = 4.16), gastrooesophageal reflux disease (n = 23, ROR = 1.73), vision blurred (n = 32, ROR = 1.5), and insomnia (n = 61, ROR = 1.46), which had not been previously reported. Moreover, other AEs listed in the voclosporin package insert, such as malignancies, QT prolongation, and pure red cell aplasia, did not meet the criteria for positive signal.

**TABLE 2:** The top 50 AEs of voclosporin ranked by the frequency at the PTs level.

SOC	PTs	Case numbers	ROR (95% Cl)
Vascular disorders	Hypertension	442	12.02 (10.93–13.23)
Nervous system disorders	Headache	404	3.79 (3.44–4.19)
Gastrointestinal disorders	Nausea	342	2.62 (2.35–2.92)
Gastrointestinal disorders	Diarrhoea	297	2.4 (2.14–2.7)
Renal and urinary disorders	Proteinuria	231	62.91 (55.13–71.79)
Infections and infestations	COVID-19	196	2.56 (2.23–2.95)
Gastrointestinal disorders	Vomiting	185	2.42 (2.1–2.8)
Gastrointestinal disorders	Abdominal discomfort	181	5.34 (4.61–6.19)
Gastrointestinal disorders	Abdominal pain upper	181	5.12 (4.42–5.93)
Investigations	Blood pressure increased	159	5.27 (4.51–6.16)
Investigations	Blood creatinine increased	148	13.79 (11.72–16.22)
Musculoskeletal and connective tissue disorders	Arthralgia	143	1.69 (1.43–1.99)
Investigations	Glomerular filtration rate decreased	143	57.83 (48.93–68.35)
Skin and subcutaneous tissue disorders	Alopecia	132	3.4 (2.86–4.04)
Renal and urinary disorders	Renal impairment	130	7.68 (6.46–9.13)
Nervous system disorders	Dizziness	129	1.59 (1.34–1.89)
Investigations	Urine protein/creatinine ratio increased	115	579.34 (471.14–712.37)
Musculoskeletal and connective tissue disorders	Systemic lupus erythematosus	109	13.24 (10.96–16)
Respiratory, thoracic and mediastinal disorders	Cough	91	1.6 (1.3–1.97)
Blood and lymphatic system disorders	Anaemia	82	2.59 (2.08–3.21)
Infections and infestations	Urinary tract infection	82	2.47 (1.99–3.07)
Investigations	Laboratory test abnormal	79	15.1 (12.09–18.85)
Musculoskeletal and connective tissue disorders	Pain in extremity	71	1.39 (1.1–1.75)
Infections and infestations	Nasopharyngitis	70	1.87 (1.48–2.37)
Gastrointestinal disorders	Dyspepsia	69	4.32 (3.41–5.47)
Metabolism and nutrition disorders	Decreased appetite	66	1.5 (1.18–1.91)
Gastrointestinal disorders	Abdominal pain	62	1.56 (1.22–2.01)
Psychiatric disorders	Insomnia	61	1.46 (1.14–1.88)
Musculoskeletal and connective tissue disorders	Joint swelling	61	2.29 (1.78–2.95)
Nervous system disorders	Migraine	60	3.25 (2.52–4.19)
Musculoskeletal and connective tissue disorders	Back pain	58	1.42 (1.1–1.84)
Musculoskeletal and connective tissue disorders	Muscle spasms	55	1.87 (1.44–2.44)
Renal and urinary disorders	Acute kidney injury	54	1.4 (1.07–1.82)
Nervous system disorders	Tremor	52	2.05 (1.56–2.7)
Infections and infestations	Infection	46	1.57 (1.18–2.1)
Infections and infestations	Influenza	46	2.1 (1.57–2.81)
Nervous system disorders	Somnolence	46	1.35 (1.01–1.81)
Gastrointestinal disorders	Gastrointestinal disorder	43	2.44 (1.81–3.29)
Infections and infestations	Herpes zoster	40	3.48 (2.55–4.74)
Metabolism and nutrition disorders	Dehydration	35	1.74 (1.25–2.43)
Investigations	Protein urine present	33	33.08 (23.44–46.67)
Renal and urinary disorders	Renal disorder	33	3.78 (2.69–5.32)
Investigations	Haemoglobin decreased	32	1.84 (1.3–2.61)
Investigations	Heart rate increased	32	1.82 (1.29–2.57)
Eye disorders	Vision blurred	32	1.5 (1.06–2.12)
Gastrointestinal disorders	Abdominal distension	29	1.64 (1.14–2.36)
Investigations	Blood pressure abnormal	27	6.35 (4.35–9.27)
Investigations	Blood potassium increased	23	9.26 (6.15–13.96)
Metabolism and nutrition disorders	Fluid retention	23	2.64 (1.76–3.98)
Gastrointestinal disorders	Gastrooesophageal reflux disease	23	1.73 (1.15–2.61)

**TABLE 3:** The top 50 signal strength of AEs of voclosporin ranked by the ROR at the PTs level.

SOC	PTs	Case numbers	ROR (95% Cl)
Investigations	Serology abnormal	5	1,183.84 (396.68–3,533.01)
Investigations	Urine protein/creatinine ratio increased	115	579.34 (471.14–712.37)
Investigations	Urine protein/creatinine ratio decreased	4	568.2 (188.55–1712.26)
Investigations	Complement factor C4 decreased	10	453.58 (229.15–897.83)
Investigations	Complement factor decreased	8	378.93 (178.59–804)
Investigations	Complement factor C3 decreased	12	376.27 (203.64–695.25)
Infections and infestations	Streptobacillus infection	3	245.83 (74.4–812.32)
Investigations	Double stranded Dna antibody positive	12	224.44 (123.78–406.96)
Investigations	Urine protein/creatinine ratio abnormal	7	167.63 (77.64–361.9)
Investigations	Urine albumin/creatinine ratio increased	9	100.97 (51.73–197.1)
Renal and urinary disorders	Proteinuria	231	62.91 (55.13–71.79)
Investigations	Glomerular filtration rate decreased	143	57.83 (48.93–68.35)
Investigations	Protein total increased	21	44.5 (28.87–68.58)
Investigations	Glomerular filtration rate abnormal	16	43.25 (26.36–70.97)
Vascular disorders	Hypertensive urgency	6	36.02 (16.07–80.73)
Vascular disorders	Malignant hypertension	3	35.51 (11.34–111.16)
Investigations	Glomerular filtration rate increased	8	35.45 (17.62–71.31)
Investigations	Protein urine	5	34.04 (14.07–82.37)
Investigations	Protein urine present	33	33.08 (23.44–46.67)
Investigations	Blood albumin abnormal	3	31.18 (9.97–97.49)
Renal and urinary disorders	Albuminuria	3	30.15 (9.64–94.24)
Investigations	Blood creatine abnormal	3	30.15 (9.64–94.24)
Infections and infestations	Pyuria	4	27.06 (10.09–72.55)
Renal and urinary disorders	Lupus nephritis	10	25.93 (13.9–48.39)
Renal and urinary disorders	Microalbuminuria	3	24.12 (7.73–75.27)
Skin and subcutaneous tissue disorders	Hypertrichosis	8	23.91 (11.91–48.02)
Skin and subcutaneous tissue disorders	Butterfly rash	5	23.84 (9.87–57.56)
Renal and urinary disorders	Urine abnormality	20	20.06 (12.91–31.17)
Investigations	Blood creatinine abnormal	13	19.8 (11.46–34.2)
Gastrointestinal disorders	Gastrointestinal tract irritation	4	18.53 (6.92–49.59)
Skin and subcutaneous tissue disorders	Hair growth abnormal	16	16 (9.78–26.17)
Investigations	Blood creatine increased	12	15.35 (8.7–27.09)
Investigations	Laboratory test abnormal	79	15.1 (12.09–18.85)
Investigations	Protein total decreased	7	14.37 (6.83–30.23)
Investigations	Blood creatinine increased	148	13.79 (11.72–16.22)
Musculoskeletal and connective tissue disorders	Systemic lupus erythematosus	109	13.24 (10.96–16)
Eye disorders	Eyelid rash	3	13.1 (4.21–40.76)
Gastrointestinal disorders	Gingival swelling	8	13.01 (6.49–26.07)
Investigations	Protein total abnormal	5	12.62 (5.24–30.41)
Gastrointestinal disorders	Breath odour	4	12.5 (4.68–33.4)
Infections and infestations	Coccidioidomycosis	3	12.32 (3.96–38.32)
Vascular disorders	Hypertension	442	12.02 (10.93–13.23)
Skin and subcutaneous tissue disorders	Skin depigmentation	3	10.94 (3.52–34.04)
Metabolism and nutrition disorders	Hypervolaemia	13	10.27 (5.96–17.72)
Investigations	Blood potassium increased	23	9.26 (6.15–13.96)
Investigations	Blood albumin decreased	9	8.63 (4.48–16.61)
Investigations	Bacterial test positive	4	8.54 (3.2–22.8)
Respiratory, thoracic and mediastinal disorders	Pleuritic pain	3	8.33 (2.68–25.9)
Investigations	Blood creatinine decreased	5	7.95 (3.3–19.14)
Investigations	Blood potassium abnormal	4	7.91 (2.96–21.13)

### Subgroup analysis of signals for preferred terms

**FIGURE 2:**
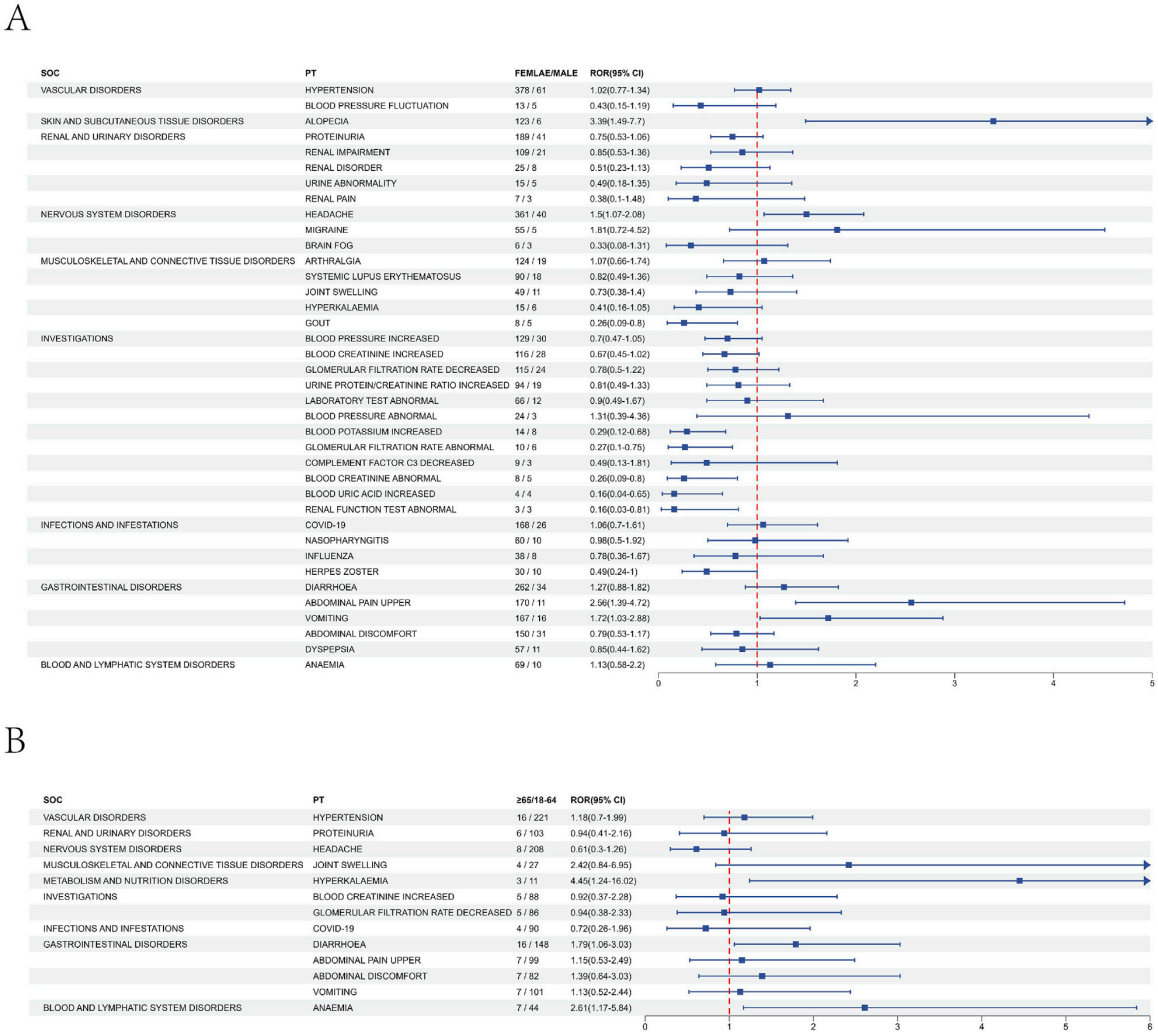
Subgroup analysis results of voclosporin adverse events. **(A)** Analysis results of the gender subgroup. **(B)** Analysis results of the age subgroup.

To analyze the gender differences in safety signals of voclosporin, we employed the ROR algorithm to screen voclosporin-related AEs separately for males and females. In males, we identified 46 AEs (Supplementary Table S3), while in females, 122 AEs were identified (Supplementary Table S4). Comparing the AEs of different genders, we can find that 38 AEs can be observed in both male and female, while certain AEs were gender-specific. The top five AEs exclusive to females were nausea, dizziness, cough, urinary tract infection, and decreased appetite. The top five AEs exclusive to males were back pain, muscle spasms, vision impairment, neck pain, and dry mouth. Among the AEs common to both sexes, alopecia, headache, upper abdominal pain, and vomiting were more strongly associated with females, whereas gout, abnormal serum creatinine levels, elevated serum uric acid, and renal function abnormalities were more strongly associated with males (Figure 2A).

We also assessed the risk differences of safety signals between adults (aged 18–64) and the elderly (aged 65 and above). Within the 18–64 age group, we identified 96 AEs (Supplementary Table S5), whereas in the group aged 65 and above, we identified 15 AEs (Supplementary Table S6). Among individuals aged 18–64, the most frequently reported top five AEs were nausea, urine protein/creatinine ratio increased, dizziness, arthralgia, and renal impairment. In contrast, AEs exclusive to those aged 65 and above included joint swelling, arthritis, fluid retention, memory impairment, hyperkalaemia, white blood cell count decreased, and dry mouth. Further analysis revealed that among the 11 shared positive PT signals in both age subgroups, patients aged 65 and above had a higher relative risk for hyperkalaemia and anaemia compared to those aged 18–64 (Figure 2B).

### Onset time of events

**FIGURE 3:**
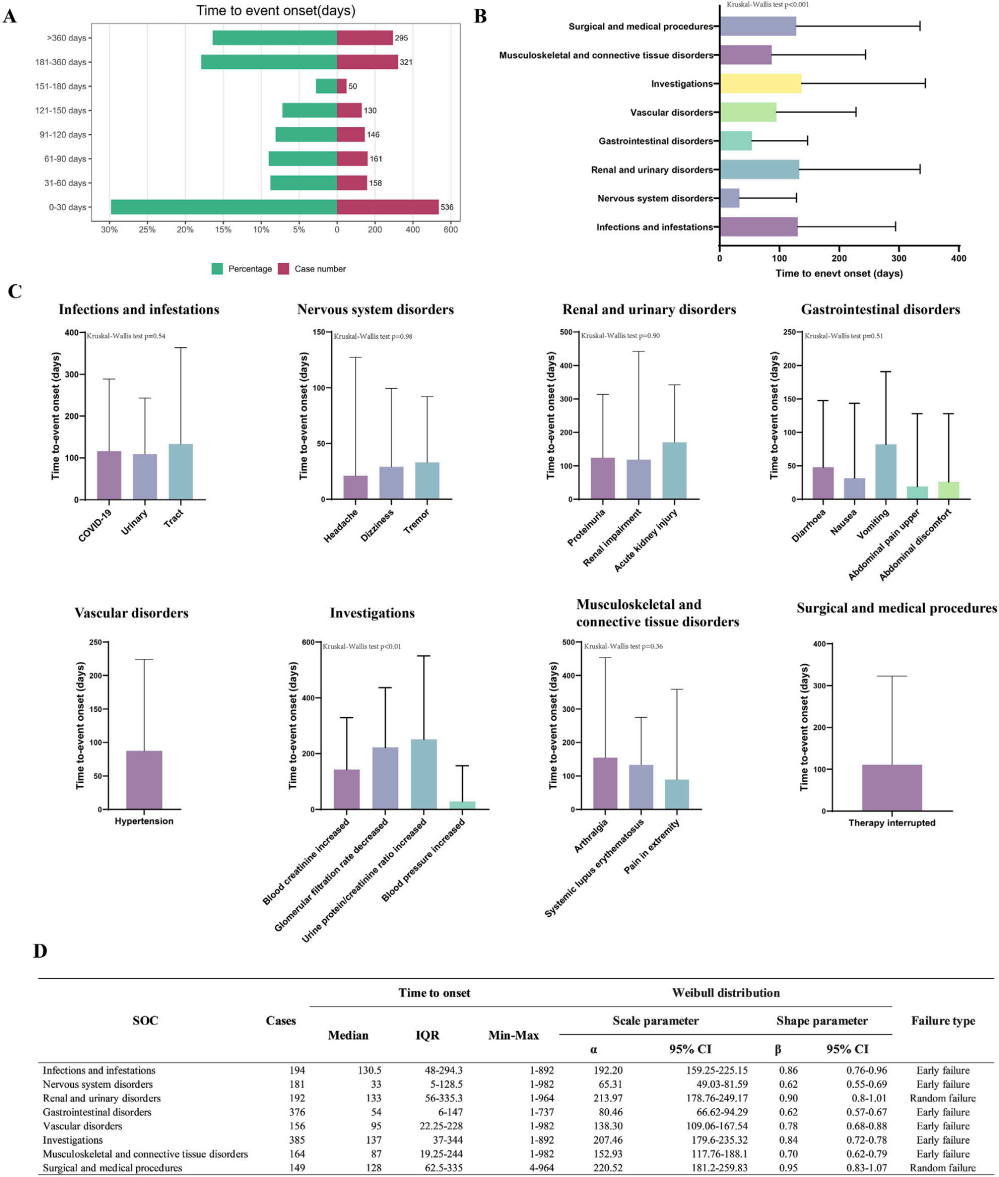
Analysis results of voclosporin-related adverse event onset times. **(A)** Overall time distribution of adverse event onset times. **(B)** Median time comparison at the SOC Level. **(C)** Median time comparison at the PT Level. **(D)** Summary of onset times at the SOC Level.

We analyzed 1,797 case reports of voclosporin-related AEs with known onset times. The median initiation period stood at 99 days (IQR 20–250). Nearly one-third of the AEs (n = 536, 29.8%) occurred within the first month of initiating voclosporin. Furthermore, 17.9% of AEs were reported between 180 and 360 days, and 16.4% occurred after 360 days (Figure 3A). Further analysis at the primary SOC level showed significant differences in the onset times of AEs among various SOCs (p < 0.01). In the reports of “renal and urinary disorders” and “infections and infestations,” the median onset times of adverse events were 133 days (IQR 56–335.3) and 130.5 days (IQR 48–294.3), respectively, which were significantly later than the median times for “gastrointestinal disorders” (median time 54 days, IQR 6–147) and “nervous system disorders” (median time 33 days, IQR 5–128.5) (Figure 3B). For clarity on the onset time of individual PTs in the SOC, we analyzed and compared the detailed onset times of AEs at the PT level according to the SOC. The Kruskal-Wallis test revealed statistically significant differences in the onset times of PT within “investigations” (P < 0.001) (Figure 3C). Specifically, the median onset time for blood pressure increased was the shortest (28, IQR 6–156.5), while that for increased urinary protein/creatinine ratio was the longest (251, IQR 109–550) (Supplementary Table S7). Ultimately, the Weibull parameter test results indicated that the onset times of adverse events in the “renal and urinary disorders” and “surgical and medical procedures” exhibited characteristics of a random failure type, while other SOCs showed an early failure type (Figure 3D).

## Discussion

At present, comprehensive real-world, large-sample safety studies on voclosporin are limited, with the majority of PubMed research focusing on small-scale, single-population studies. This study represented the first systematic pharmacovigilance research using the FAERS database on the safety of voclosporin following its market introduction.

### Baseline data description

Our study indicated that the proportion of AEs was higher in females (84%) compared to males (14.8%) and a higher proportion aged between 18 and 44 years (26.4%). This finding aligns with epidemiological studies on LN, which reported that the prevalence of LN in females is approximately three to four times that in males, with a median age at diagnosis of about 38.4 years (Hocaoǧlu et al., 2023; Feldman et al., 2013). Notably, only 20% of reports contained clear outcome data, potentially due to a significant number of reports being submitted by consumers, which may have led to underreporting of outcome information. This highlights the need for increased vigilance among clinicians and pharmacists regarding voclosporin-related AEs. In addition, among the available outcome reports, more than 50% indicated serious adverse events (hospitalization, death, disability). Although this may be attributed to the severity or progression of the underlying condition, it still warrants attention from clinicians. Regarding reporting by country, the number of AEs in the United States significantly surpassed those in other countries, likely due to the earlier availability and higher prescription volume of the drug in the country. Given that the mechanisms and effectiveness of therapeutic interventions for LN vary among racial and ethnic subgroups (Isenberg et al., 2010), our analytical findings might differ in other regions. Thus, studies across different ethnicities and regions are required to validate our findings.

### Risk of renal toxicity

Voclosporin is the first oral agent approved for the treatment of LN that was developed through structural modification of CsA. Like CsA, voclosporin inhibits T-cell activation by suppressing calcineurin. However, structural modification endows voclosporin with stronger immunosuppressive effects (Kuglstatter et al., 2011), which has been confirmed *in vivo* using a 32P-labelled calcineurin activity assay (Stalder et al., 2003). When compared to another CNI, tacrolimus, voclosporin also demonstrated comparable immunosuppressive efficacy in preventing renal transplant rejection (Anglade et al., 2008). Although voclosporin is associated with a lower metabolite burden associated with nephrotoxicity compared to CsA (Roby and Shaw, 1993; Ling et al., 2014), the AEs remain inevitable. In our study, we observed a substantial number of nephrotoxicity-related AEs, such as blood creatinine increased (n = 148, ROR = 13.79), eGFR reduced (n = 143, ROR = 57.83), and acute kidney injury (n = 54, ROR = 1.4). Therefore, during voclosporin treatment, regular monitoring of renal function is necessary to adjust the dosage based on the eGFR.

### Risk of hypertension

Hypertension is common in patients treated with voclosporin. Integrated analysis of published AURA-LV and AURORA 1 trials revealed that up to 19.1% of LN patients receiving voclosporin experienced hypertension (Arriens et al., 2023). This finding corroborated our study, where hypertension (n = 442, ROR = 12.02) was the most frequently reported AE. Hypertension is also a relatively common AE among other CNI. The underlying mechanisms are believed to include activation of the renin-angiotensin system and the sympathetic nervous system, induction of oxidative stress, and alteration of the nitric oxide (NO) signaling pathway, leading to systemic vasoconstriction and elevated blood pressure (Hošková et al., 2017). Blood pressure abnormalities during voclosporin treatment are generally transient, and no patients had treatment discontinued due to hypertension (Arriens et al., 2023). Nevertheless, monitoring blood pressure remains necessary during treatment. Some calcium channel blockers (verapamil and diltiazem) may increase voclosporin blood concentrations (Ling et al., 2014), necessitating a reduction in the dose of voclosporin. If elevated blood pressure is not controlled by a reduction in voclosporin dosage alone, further medical interventions may be required. Besides hypertension, diabetes is also one of the classically CNI-attributed complications. In our study, no positive AEs related to diabetes were observed, and several other studies also proved the superiority of voclosporin in this regard (Rovin et al., 2019; Rovin et al., 2021; Kolic et al., 2020; Busque et al., 2011). Voclosporin has a milder impact on pancreatic islet function compared to other CNIs, which address the clinical needs of diabetes patients for CNI therapy.

### Risk of infections and malignancies

Voclosporin’s prescribing information contains a black box warning about the increased risk of serious infection and malignancies, potentially leading to hospitalization or death, which is one of the unavoidable side effects of immunosuppressants (Chua et al., 2020). In the AURA-LV and AURORA 1 randomized controlled trials, infections were the most common AEs in both the voclosporin group and the placebo group, with 40 cases (15%) and 30 cases (11.3%) of patients considered treatment-related. Infection was also considered as the positive signal in our study, including COVID-19 (n = 196, ROR = 2.56) and urinary tract infection (n = 82, ROR = 2.47). Malignancies did not meet the positive criteria for this study. Patients treated with immunomodulatory agents are generally at an increased risk of developing malignant tumors (Munagala and Phancao, 2022; Prakash et al., 2002), with risk factors including male gender and advanced age (Ilham et al., 2023; Pendón-Ruiz de Mier et al., 2015). However, these factors differ from the epidemiological characteristics of LN (). Moreover, the relatively short time since the market introduction may also explain the negative signal for malignant tumors observed in this study. Notably, multiple studies have pointed out that compared to other immunosuppressants, CNIs were associated more with the development of malignancies (Pendón-Ruiz de Mier et al., 2015; Doesch et al., 2010; Kulbat et al., 2023). Therefore, it is recommended that healthcare professionals continue vigilant monitoring for malignant AEs during the clinical use of voclosporin.

### Adverse events related to primary disease

In our study, positive SOC signals related to the primary disease were also reported, including “musculoskeletal and connective tissue disorders” and “investigations.” LN is secondary to SLE. The numerous positive AEs observed in this study, such as arthralgia (n = 143, ROR = 1.69), pain in extremity (n = 71, ROR = 1.39), malar rash (n = 109, ROR = 13.24), and proteinuria detection (n = 33, ROR = 33.08), represented the different organ manifestations of SLE (Kiriakidou and Ching, 2020). Given that 80% of the reports in this study were submitted by consumers, we have reason to believe that, although LN is not a rare disease, it remains poorly understood. This highlights the necessity for patients on voclosporin to be well-informed about the manifestations of LN progression and to distinguish them from drug adverse reactions.

### Adverse events at other SOC level

Among the SOC of nervous system disorders, a large number of AEs were also reported, such as headaches (n = 404, ROR = 3.79), dizziness (n = 129, ROR = 1.59), and migraine (n = 60, ROR = 3.25). This could be attributable to the neurotoxicity of CNIs (Faravelli et al., 2021), as described in the product instructions. Previous studies found that the incidence of gastrointestinal-related AEs was higher in the treatment group compared with the placebo group (Rovin et al., 2021). In our study, gastrointestinal-related AEs (n = 1737, ROR = 2.03) notably exceeded expectations and represented the highest number of AEs across all SOC. Common AEs included nausea (n = 342, ROR = 2.62), diarrhea (n = 297, ROR = 2.4), and vomiting (n = 185, ROR = 2.42). Although not being explicitly detailed in the product instruction, it is necessary for physicians to inform patients about the potential risk of gastrointestinal-related AEs. Additionally, our findings revealed some rare and unexpected AEs including breath odor (n = 4, ROR = 12.5), gastroesophageal reflux disease (n = 23, ROR = 1.73), menstrual disorder (n = 7, ROR = 5.82), vision blurred (n = 32, ROR = 1.5), insomnia (n = 61, ROR = 1.46), and acute pulmonary oedema (n = 3, ROR = 4.16). These adverse reactions require further research to elucidate their specific mechanisms of occurrence.

### Gender and age differences in adverse events

Gender differences have been shown to influence drug bioavailability, distribution, metabolism, and elimination (Zopf et al., 2009), leading to disparities in ADEs between males and females. However, there is a lack of reports on gender-specific ADEs associated with voclosporin treatment. In our study, we observed a greater number of positive signal values for ADEs in females compared to males. Alopecia, headache, upper abdominal pain, and vomiting exhibited a stronger correlation with females, while gout, abnormal serum creatinine levels, increased serum uric acid, and abnormal renal function tests demonstrated a stronger correlation with males. Some of these adverse reactions are inherently gender-specific; for example, the incidence of headache was higher in females than in males (Silberstein, 2000). Serum uric acid levels tend to increase steadily in males before the age of 50, while levels remain stable in females (Zitt et al., 2020). This might have been the reason why the uric acid metabolism in males was more susceptible to the effects of medications. In the age subgroup analysis, we found that older adults had a higher relative risk of hyperkalemia and anemia compared to younger adults. Hyperkalemia is a common electrolyte disturbance induced by CNIs, which can lead to life-threatening arrhythmias. This ionic disturbance is more challenging to regulate due to the presence of underlying kidney disease in patients. A strong positive correlation exists between the severity of hyperkalemia and mortality risk (Sun et al., 2024), making early identification and treatment of hyperkalemia crucial. Healthcare providers need to implement interventions to enhance patient awareness of hyperkalemia. In summary, our findings provide insights into population-specific side effects. While these findings require further validation, they offer improved guidance for drug monitoring in diverse populations.

### Time to onset of adverse events

In this study, 31.35% of AEs associated with voclosporin occurred within the first month of treatment, notably gastrointestinal and neurological AEs; the total number of AEs decreased monthly thereafter. At the specific SOC level, the median reporting times for renal and infectious AEs were observed to be later. In summary, clinical monitoring strategies should continue to be optimized, with a particular emphasis on the early detection and management, especially during the first month of voclosporin treatment. Furthermore, heightened vigilance is required for renal and infectious adverse reactions during prolonged therapy.

### Limitations

This study was subject to several limitations. Firstly, the FAERS database is an open, voluntary reporting system. The AE information might be delayed, incomplete, and inaccurate due to the different reporters (professionals and non-professionals). For example, 60.5% of the reports in this study did not provide age information and 80.7% did not provide treatment outcomes. Second, voclosporin often combines with other immunosuppressive drugs. The FDA analyzes all collected adverse drug events, which may confound some adverse reactions, as they might be the result of combination therapy or different treatment strategies. Moreover, our research could only provide a statistical association between voclosporin and AEs, the true incidence of each AE could not be calculated due to the absence of data on the total number of cases occurring in the population. Therefore, while the research provides valuable insights, further investigation is still required to get a comprehensive understanding of the risk associated with voclosporin use.

## Conclusion

The present study conducted a comprehensive pharmacovigilance analysis of voclosporin based on the FAERS database. Our study has unearthed numerous voclosporin safety signals consistent with clinical trials, such as “renal and urinary disorders” and “infections and infestations.” Notably, the number of gastrointestinal disease-related AEs reported exceeded expectations and were not mentioned in the product instruction, necessitating further evaluation. These findings provided valuable insights into the safety profiles of voclosporin in real-world practice.

## Data Availability

The original contributions presented in the study are included in the article/Supplementary Material, further inquiries can be directed to the corresponding author.
