# Supplementary material for: Post-marketing safety surveillance of voclosporin: an observational, pharmacovigilance study leveraging faers database study on the safety of voclosporin
Source: Front Pharmacol. 2025 May 19;16:1506760. doi: 10.3389/fphar.2025.1506760 (PMC12128604; doi:10.3389/fphar.2025.1506760)
Supplement: Supplementary file 1 [file Table1.doc]

**Supplementary Table S1** (A) Fourfold table of disproportionality analyses. (B) ROR method

A Fourfold table of disproportionality analyses.

| Medicine | Target adverse events reported | Other adverse events reported | Summation |
| --- | --- | --- | --- |
| Target drugs | a | b | a+b |
| Other drugs | c | d | c + d |
| Summation | a+c | b + d | a+b + c + d |

a, Number of reports containing both voclosporin and the specified adverse event; b, Number of reports containing voclosporin and adverse events other than the specified event; c, Number of reports containing other drugs and the specified adverse event; d, Number of reports containing other drugs and adverse events other than the specified event; Other drugs, all drugs in the FAERS dataset excluding voclosporin.

B ROR method

| Algorithms | Equation |
| --- | --- |
| ROR | ROR = ad/bc 95%CI = eln(ROR)±1.96(1/a+1/b+1/c+1/d) ^ 0.5 |

**Supplementary Table S2** All positive AEs associated with voclosporin in the FAERS database.

| **SOC** | **PTs** | **Case numbers** | **ROR(95%Cl)** |
| --- | --- | --- | --- |
| Vascular Disorders | Hypertension | 442 | 12.02 ( 10.93 - 13.23 ) |
| Nervous System Disorders | Headache | 404 | 3.79 ( 3.44 - 4.19 ) |
| Gastrointestinal Disorders | Nausea | 342 | 2.62 ( 2.35 - 2.92 ) |
| Gastrointestinal Disorders | Diarrhoea | 297 | 2.4 ( 2.14 - 2.7 ) |
| Renal And Urinary Disorders | Proteinuria | 231 | 62.91 ( 55.13 - 71.79 ) |
| Infections And Infestations | Covid-19 | 196 | 2.56 ( 2.23 - 2.95 ) |
| Gastrointestinal Disorders | Vomiting | 185 | 2.42 ( 2.1 - 2.8 ) |
| Gastrointestinal Disorders | Abdominal Pain Upper | 181 | 5.12 ( 4.42 - 5.93 ) |
| Gastrointestinal Disorders | Abdominal Discomfort | 181 | 5.34 ( 4.61 - 6.19 ) |
| Investigations | Blood Pressure Increased | 159 | 5.27 ( 4.51 - 6.16 ) |
| Investigations | Blood Creatinine Increased | 148 | 13.79 ( 11.72 - 16.22 ) |
| Investigations | Glomerular Filtration Rate Decreased | 143 | 57.83 ( 48.93 - 68.35 ) |
| Musculoskeletal And Connective Tissue Disorders | Arthralgia | 143 | 1.69 ( 1.43 - 1.99 ) |
| Skin And Subcutaneous Tissue Disorders | Alopecia | 132 | 3.4 ( 2.86 - 4.04 ) |
| Renal And Urinary Disorders | Renal Impairment | 130 | 7.68 ( 6.46 - 9.13 ) |
| Nervous System Disorders | Dizziness | 129 | 1.59 ( 1.34 - 1.89 ) |
| Investigations | Urine Protein/Creatinine Ratio Increased | 115 | 579.34 ( 471.14 - 712.37 ) |
| Musculoskeletal And Connective Tissue Disorders | Systemic Lupus Erythematosus | 109 | 13.24 ( 10.96 - 16 ) |
| Respiratory, Thoracic And Mediastinal Disorders | Cough | 91 | 1.6 ( 1.3 - 1.97 ) |
| Blood And Lymphatic System Disorders | Anaemia | 82 | 2.59 ( 2.08 - 3.21 ) |
| Infections And Infestations | Urinary Tract Infection | 82 | 2.47 ( 1.99 - 3.07 ) |
| Investigations | Laboratory Test Abnormal | 79 | 15.1 ( 12.09 - 18.85 ) |
| Musculoskeletal And Connective Tissue Disorders | Pain In Extremity | 71 | 1.39 ( 1.1 - 1.75 ) |
| Infections And Infestations | Nasopharyngitis | 70 | 1.87 ( 1.48 - 2.37 ) |
| Gastrointestinal Disorders | Dyspepsia | 69 | 4.32 ( 3.41 - 5.47 ) |
| Metabolism And Nutrition Disorders | Decreased Appetite | 66 | 1.5 ( 1.18 - 1.91 ) |
| Gastrointestinal Disorders | Abdominal Pain | 62 | 1.56 ( 1.22 - 2.01 ) |
| Psychiatric Disorders | Insomnia | 61 | 1.46 ( 1.14 - 1.88 ) |
| Musculoskeletal And Connective Tissue Disorders | Joint Swelling | 61 | 2.29 ( 1.78 - 2.95 ) |
| Nervous System Disorders | Migraine | 60 | 3.25 ( 2.52 - 4.19 ) |
| Musculoskeletal And Connective Tissue Disorders | Back Pain | 58 | 1.42 ( 1.1 - 1.84 ) |
| Musculoskeletal And Connective Tissue Disorders | Muscle Spasms | 55 | 1.87 ( 1.44 - 2.44 ) |
| Renal And Urinary Disorders | Acute Kidney Injury | 54 | 1.4 ( 1.07 - 1.82 ) |
| Nervous System Disorders | Tremor | 52 | 2.05 ( 1.56 - 2.7 ) |
| Nervous System Disorders | Somnolence | 46 | 1.35 ( 1.01 - 1.81 ) |
| Infections And Infestations | Infection | 46 | 1.57 ( 1.18 - 2.1 ) |
| Infections And Infestations | Influenza | 46 | 2.1 ( 1.57 - 2.81 ) |
| Gastrointestinal Disorders | Gastrointestinal Disorder | 43 | 2.44 ( 1.81 - 3.29 ) |
| Infections And Infestations | Herpes Zoster | 40 | 3.48 ( 2.55 - 4.74 ) |
| Metabolism And Nutrition Disorders | Dehydration | 35 | 1.74 ( 1.25 - 2.43 ) |
| Investigations | Protein Urine Present | 33 | 33.08 ( 23.44 - 46.67 ) |
| Renal And Urinary Disorders | Renal Disorder | 33 | 3.78 ( 2.69 - 5.32 ) |
| Investigations | Haemoglobin Decreased | 32 | 1.84 ( 1.3 - 2.61 ) |
| Investigations | Heart Rate Increased | 32 | 1.82 ( 1.29 - 2.57 ) |
| Eye Disorders | Vision Blurred | 32 | 1.5 ( 1.06 - 2.12 ) |
| Gastrointestinal Disorders | Abdominal Distension | 29 | 1.64 ( 1.14 - 2.36 ) |
| Investigations | Blood Pressure Abnormal | 27 | 6.35 ( 4.35 - 9.27 ) |
| Metabolism And Nutrition Disorders | Fluid Retention | 23 | 2.64 ( 1.76 - 3.98 ) |
| Gastrointestinal Disorders | Gastrooesophageal Reflux Disease | 23 | 1.73 ( 1.15 - 2.61 ) |
| Investigations | Blood Potassium Increased | 23 | 9.26 ( 6.15 - 13.96 ) |
| Investigations | Protein Total Increased | 21 | 44.5 ( 28.87 - 68.58 ) |
| Respiratory, Thoracic And Mediastinal Disorders | Epistaxis | 21 | 1.68 ( 1.09 - 2.57 ) |
| Metabolism And Nutrition Disorders | Hyperkalaemia | 21 | 3.81 ( 2.48 - 5.84 ) |
| Renal And Urinary Disorders | Urine Abnormality | 20 | 20.06 ( 12.91 - 31.17 ) |
| Renal And Urinary Disorders | Pollakiuria | 18 | 2.63 ( 1.66 - 4.18 ) |
| Gastrointestinal Disorders | Flatulence | 18 | 1.89 ( 1.19 - 3 ) |
| Vascular Disorders | Blood Pressure Fluctuation | 18 | 2.74 ( 1.73 - 4.36 ) |
| Skin And Subcutaneous Tissue Disorders | Hair Growth Abnormal | 16 | 16 ( 9.78 - 26.17 ) |
| Investigations | Glomerular Filtration Rate Abnormal | 16 | 43.25 ( 26.36 - 70.97 ) |
| Infections And Infestations | Gastroenteritis Viral | 15 | 4.39 ( 2.64 - 7.28 ) |
| Investigations | Blood Urine Present | 15 | 5 ( 3.01 - 8.3 ) |
| Gastrointestinal Disorders | Frequent Bowel Movements | 14 | 2.22 ( 1.32 - 3.76 ) |
| Investigations | Blood Test Abnormal | 13 | 3.82 ( 2.22 - 6.59 ) |
| Metabolism And Nutrition Disorders | Gout | 13 | 4.09 ( 2.37 - 7.05 ) |
| Metabolism And Nutrition Disorders | Hypervolaemia | 13 | 10.27 ( 5.96 - 17.72 ) |
| Investigations | Blood Creatinine Abnormal | 13 | 19.8 ( 11.46 - 34.2 ) |
| Investigations | Blood Urea Increased | 13 | 7.25 ( 4.21 - 12.5 ) |
| Musculoskeletal And Connective Tissue Disorders | Joint Stiffness | 12 | 2.46 ( 1.4 - 4.33 ) |
| Renal And Urinary Disorders | Dysuria | 12 | 2 ( 1.14 - 3.52 ) |
| Investigations | Double Stranded Dna Antibody Positive | 12 | 224.44 ( 123.78 - 406.96 ) |
| Investigations | Blood Creatine Increased | 12 | 15.35 ( 8.7 - 27.09 ) |
| Investigations | Complement Factor C3 Decreased | 12 | 376.27 ( 203.64 - 695.25 ) |
| Infections And Infestations | Kidney Infection | 11 | 2.64 ( 1.46 - 4.77 ) |
| Renal And Urinary Disorders | Renal Pain | 10 | 5.35 ( 2.87 - 9.95 ) |
| Renal And Urinary Disorders | Lupus Nephritis | 10 | 25.93 ( 13.9 - 48.39 ) |
| Investigations | Complement Factor C4 Decreased | 10 | 453.58 ( 229.15 - 897.83 ) |
| Gastrointestinal Disorders | Mouth Ulceration | 10 | 2.54 ( 1.37 - 4.72 ) |
| Reproductive System And Breast Disorders | Heavy Menstrual Bleeding | 10 | 4.59 ( 2.47 - 8.54 ) |
| Investigations | Blood Albumin Decreased | 9 | 8.63 ( 4.48 - 16.61 ) |
| Investigations | Urine Albumin/Creatinine Ratio Increased | 9 | 100.97 ( 51.73 - 197.1 ) |
| Nervous System Disorders | Brain Fog | 9 | 3.09 ( 1.61 - 5.94 ) |
| Skin And Subcutaneous Tissue Disorders | Hypertrichosis | 8 | 23.91 ( 11.91 - 48.02 ) |
| Investigations | Blood Uric Acid Increased | 8 | 7.63 ( 3.81 - 15.27 ) |
| Investigations | Blood Iron Decreased | 8 | 2.91 ( 1.45 - 5.82 ) |
| Gastrointestinal Disorders | Gingival Swelling | 8 | 13.01 ( 6.49 - 26.07 ) |
| Gastrointestinal Disorders | Gingival Bleeding | 8 | 3.88 ( 1.94 - 7.76 ) |
| Investigations | Complement Factor Decreased | 8 | 378.93 ( 178.59 - 804 ) |
| Investigations | Glomerular Filtration Rate Increased | 8 | 35.45 ( 17.62 - 71.31 ) |
| Skin And Subcutaneous Tissue Disorders | Photosensitivity Reaction | 7 | 2.37 ( 1.13 - 4.97 ) |
| Musculoskeletal And Connective Tissue Disorders | Flank Pain | 7 | 4.37 ( 2.08 - 9.17 ) |
| Investigations | Protein Total Decreased | 7 | 14.37 ( 6.83 - 30.23 ) |
| Reproductive System And Breast Disorders | Menstrual Disorder | 7 | 5.82 ( 2.77 - 12.22 ) |
| Renal And Urinary Disorders | Chromaturia | 7 | 2.14 ( 1.02 - 4.5 ) |
| Investigations | Urine Protein/Creatinine Ratio Abnormal | 7 | 167.63 ( 77.64 - 361.9 ) |
| Renal And Urinary Disorders | Polyuria | 6 | 4.12 ( 1.85 - 9.18 ) |
| Investigations | Renal Function Test Abnormal | 6 | 6.26 ( 2.81 - 13.94 ) |
| Renal And Urinary Disorders | Urine Odour Abnormal | 6 | 6 ( 2.69 - 13.38 ) |
| Musculoskeletal And Connective Tissue Disorders | Muscle Tightness | 6 | 2.39 ( 1.07 - 5.32 ) |
| Infections And Infestations | Laryngitis | 6 | 3.12 ( 1.4 - 6.95 ) |
| Metabolism And Nutrition Disorders | Iron Deficiency | 6 | 5.76 ( 2.58 - 12.84 ) |
| Vascular Disorders | Hypertensive Urgency | 6 | 36.02 ( 16.07 - 80.73 ) |
| Vascular Disorders | Raynaud'S Phenomenon | 6 | 6.68 ( 3 - 14.89 ) |
| Blood And Lymphatic System Disorders | Increased Tendency To Bruise | 5 | 3.35 ( 1.39 - 8.05 ) |
| Skin And Subcutaneous Tissue Disorders | Butterfly Rash | 5 | 23.84 ( 9.87 - 57.56 ) |
| Gastrointestinal Disorders | Faeces Soft | 5 | 2.48 ( 1.03 - 5.96 ) |
| Metabolism And Nutrition Disorders | Appetite Disorder | 5 | 3.74 ( 1.55 - 8.99 ) |
| Investigations | Protein Total Abnormal | 5 | 12.62 ( 5.24 - 30.41 ) |
| Nervous System Disorders | Posterior Reversible Encephalopathy Syndrome | 5 | 2.81 ( 1.17 - 6.75 ) |
| Investigations | Protein Urine | 5 | 34.04 ( 14.07 - 82.37 ) |
| Investigations | Blood Creatinine Decreased | 5 | 7.95 ( 3.3 - 19.14 ) |
| Investigations | Serology Abnormal | 5 | 1183.84 ( 396.68 - 3533.01 ) |
| Gastrointestinal Disorders | Breath Odour | 4 | 12.5 ( 4.68 - 33.4 ) |
| Investigations | Blood Potassium Abnormal | 4 | 7.91 ( 2.96 - 21.13 ) |
| Gastrointestinal Disorders | Gastrointestinal Tract Irritation | 4 | 18.53 ( 6.92 - 49.59 ) |
| Renal And Urinary Disorders | Nephrotic Syndrome | 4 | 3.48 ( 1.31 - 9.29 ) |
| Skin And Subcutaneous Tissue Disorders | Onychoclasis | 4 | 2.82 ( 1.06 - 7.52 ) |
| Skin And Subcutaneous Tissue Disorders | Pigmentation Disorder | 4 | 3.31 ( 1.24 - 8.83 ) |
| Infections And Infestations | Pyuria | 4 | 27.06 ( 10.09 - 72.55 ) |
| Infections And Infestations | Furuncle | 4 | 2.81 ( 1.05 - 7.49 ) |
| Investigations | Bacterial Test Positive | 4 | 8.54 ( 3.2 - 22.8 ) |
| Renal And Urinary Disorders | Nephritis | 4 | 6.2 ( 2.32 - 16.55 ) |
| Investigations | Urine Protein/Creatinine Ratio Decreased | 4 | 568.2 ( 188.55 - 1712.26 ) |
| Blood And Lymphatic System Disorders | Iron Deficiency Anaemia | 4 | 2.74 ( 1.03 - 7.3 ) |
| Infections And Infestations | Gingivitis | 3 | 3.3 ( 1.06 - 10.26 ) |
| Blood And Lymphatic System Disorders | White Blood Cell Disorder | 3 | 5.53 ( 1.78 - 17.17 ) |
| Respiratory, Thoracic And Mediastinal Disorders | Acute Pulmonary Oedema | 3 | 4.16 ( 1.34 - 12.9 ) |
| Eye Disorders | Eyelid Rash | 3 | 13.1 ( 4.21 - 40.76 ) |
| Investigations | Blood Albumin Abnormal | 3 | 31.18 ( 9.97 - 97.49 ) |
| Respiratory, Thoracic And Mediastinal Disorders | Pleuritic Pain | 3 | 8.33 ( 2.68 - 25.9 ) |
| Vascular Disorders | Malignant Hypertension | 3 | 35.51 ( 11.34 - 111.16 ) |
| Infections And Infestations | Coccidioidomycosis | 3 | 12.32 ( 3.96 - 38.32 ) |
| Renal And Urinary Disorders | Microalbuminuria | 3 | 24.12 ( 7.73 - 75.27 ) |
| Renal And Urinary Disorders | Albuminuria | 3 | 30.15 ( 9.64 - 94.24 ) |
| Infections And Infestations | Streptobacillus Infection | 3 | 245.83 ( 74.4 - 812.32 ) |
| Skin And Subcutaneous Tissue Disorders | Skin Depigmentation | 3 | 10.94 ( 3.52 - 34.04 ) |
| Musculoskeletal And Connective Tissue Disorders | Joint Lock | 3 | 5.95 ( 1.92 - 18.48 ) |
| Investigations | Blood Creatine Abnormal | 3 | 30.15 ( 9.64 - 94.24 ) |
| Infections And Infestations | Escherichia Urinary Tract Infection | 3 | 4.19 ( 1.35 - 13.02 ) |

**Supplementary Table S3** Positive signals of voclosporin adverse events in males

| **SOC** | **PTs** | **Case numbers** | **ROR(95%Cl)** |
| --- | --- | --- | --- |
| Vascular Disorders | Hypertension | 61 | 11.45 ( 8.87 - 14.79 ) |
| Renal And Urinary Disorders | Proteinuria | 41 | 65.38 ( 47.89 - 89.24 ) |
| Nervous System Disorders | Headache | 40 | 3.26 ( 2.39 - 4.47 ) |
| Gastrointestinal Disorders | Diarrhoea | 34 | 1.95 ( 1.39 - 2.73 ) |
| Gastrointestinal Disorders | Abdominal Discomfort | 31 | 7.68 ( 5.38 - 10.96 ) |
| Investigations | Blood Pressure Increased | 30 | 7.45 ( 5.19 - 10.69 ) |
| Investigations | Blood Creatinine Increased | 28 | 12.66 ( 8.71 - 18.4 ) |
| Infections And Infestations | Covid-19 | 26 | 4.13 ( 2.8 - 6.08 ) |
| Investigations | Glomerular Filtration Rate Decreased | 24 | 59.3 ( 39.57 - 88.86 ) |
| Renal And Urinary Disorders | Renal Impairment | 21 | 7.04 ( 4.58 - 10.83 ) |
| Investigations | Urine Protein/Creatinine Ratio Increased | 19 | 907.72 ( 564.26 - 1460.24 ) |
| Musculoskeletal And Connective Tissue Disorders | Arthralgia | 19 | 2.05 ( 1.3 - 3.22 ) |
| Musculoskeletal And Connective Tissue Disorders | Systemic Lupus Erythematosus | 18 | 94.25 ( 59.08 - 150.36 ) |
| Gastrointestinal Disorders | Vomiting | 16 | 1.68 ( 1.03 - 2.75 ) |
| Musculoskeletal And Connective Tissue Disorders | Back Pain | 12 | 2.11 ( 1.2 - 3.73 ) |
| Investigations | Laboratory Test Abnormal | 12 | 11.7 ( 6.63 - 20.65 ) |
| Musculoskeletal And Connective Tissue Disorders | Muscle Spasms | 11 | 2.63 ( 1.45 - 4.75 ) |
| Gastrointestinal Disorders | Abdominal Pain Upper | 11 | 2.49 ( 1.37 - 4.5 ) |
| Gastrointestinal Disorders | Dyspepsia | 11 | 5.33 ( 2.95 - 9.65 ) |
| Musculoskeletal And Connective Tissue Disorders | Joint Swelling | 11 | 4.48 ( 2.47 - 8.1 ) |
| Infections And Infestations | Nasopharyngitis | 10 | 2.27 ( 1.22 - 4.23 ) |
| Blood And Lymphatic System Disorders | Anaemia | 10 | 1.89 ( 1.01 - 3.52 ) |
| Infections And Infestations | Herpes Zoster | 10 | 8.03 ( 4.31 - 14.95 ) |
| Eye Disorders | Visual Impairment | 9 | 2.93 ( 1.52 - 5.64 ) |
| Renal And Urinary Disorders | Renal Disorder | 8 | 5.24 ( 2.62 - 10.51 ) |
| Investigations | Blood Potassium Increased | 8 | 14.69 ( 7.33 - 29.44 ) |
| Infections And Infestations | Influenza | 8 | 2.85 ( 1.42 - 5.71 ) |
| Skin And Subcutaneous Tissue Disorders | Alopecia | 6 | 3.64 ( 1.63 - 8.12 ) |
| Investigations | Glomerular Filtration Rate Abnormal | 6 | 97.32 ( 43.46 - 217.94 ) |
| Musculoskeletal And Connective Tissue Disorders | Neck Pain | 6 | 4.98 ( 2.23 - 11.1 ) |
| Gastrointestinal Disorders | Dry Mouth | 6 | 3.66 ( 1.64 - 8.17 ) |
| Metabolism And Nutrition Disorders | Hyperkalaemia | 6 | 4.61 ( 2.07 - 10.28 ) |
| Renal And Urinary Disorders | Urine Abnormality | 5 | 43.21 ( 17.92 - 104.19 ) |
| Metabolism And Nutrition Disorders | Gout | 5 | 6.19 ( 2.57 - 14.9 ) |
| Nervous System Disorders | Migraine | 5 | 4.65 ( 1.93 - 11.18 ) |
| Vascular Disorders | Blood Pressure Fluctuation | 5 | 7.06 ( 2.93 - 17 ) |
| Gastrointestinal Disorders | Gastrooesophageal Reflux Disease | 5 | 3.08 ( 1.28 - 7.41 ) |
| Investigations | Blood Creatinine Abnormal | 5 | 42.3 ( 17.55 - 102 ) |
| Investigations | Blood Uric Acid Increased | 4 | 18.85 ( 7.06 - 50.33 ) |
| Skin And Subcutaneous Tissue Disorders | Photosensitivity Reaction | 3 | 6.57 ( 2.12 - 20.41 ) |
| Investigations | Renal Function Test Abnormal | 3 | 20.31 ( 6.53 - 63.11 ) |
| Renal And Urinary Disorders | Renal Pain | 3 | 9.9 ( 3.19 - 30.76 ) |
| Investigations | Blood Pressure Abnormal | 3 | 5.08 ( 1.64 - 15.79 ) |
| Skin And Subcutaneous Tissue Disorders | Skin Disorder | 3 | 3.33 ( 1.07 - 10.33 ) |
| Nervous System Disorders | Brain Fog | 3 | 16.45 ( 5.29 - 51.12 ) |
| Investigations | Complement Factor C3 Decreased | 3 | 619.61 ( 191.68 - 2002.94 ) |

**Supplementary Table S4** Positive signals of voclosporin adverse events in females.

| **SOC** | **PTs** | **Case numbers** | **ROR(95%Cl)** |
| --- | --- | --- | --- |
| Vascular Disorders | Hypertension | 378 | 11.64 ( 10.5 - 12.9 ) |
| Nervous System Disorders | Headache | 361 | 2.96 ( 2.66 - 3.29 ) |
| Gastrointestinal Disorders | Nausea | 324 | 2.23 ( 2 - 2.49 ) |
| Gastrointestinal Disorders | Diarrhoea | 262 | 2.3 ( 2.04 - 2.6 ) |
| Renal And Urinary Disorders | Proteinuria | 189 | 89.02 ( 76.88 - 103.07 ) |
| Gastrointestinal Disorders | Abdominal Pain Upper | 170 | 4.53 ( 3.89 - 5.27 ) |
| Infections And Infestations | Covid-19 | 168 | 4.22 ( 3.63 - 4.92 ) |
| Gastrointestinal Disorders | Vomiting | 167 | 2.08 ( 1.78 - 2.42 ) |
| Gastrointestinal Disorders | Abdominal Discomfort | 150 | 4.54 ( 3.86 - 5.33 ) |
| Investigations | Blood Pressure Increased | 129 | 5.1 ( 4.29 - 6.07 ) |
| Musculoskeletal And Connective Tissue Disorders | Arthralgia | 124 | 1.52 ( 1.27 - 1.81 ) |
| Skin And Subcutaneous Tissue Disorders | Alopecia | 123 | 2.47 ( 2.07 - 2.95 ) |
| Investigations | Blood Creatinine Increased | 116 | 17.06 ( 14.2 - 20.5 ) |
| Investigations | Glomerular Filtration Rate Decreased | 115 | 74 ( 61.4 - 89.19 ) |
| Nervous System Disorders | Dizziness | 115 | 1.34 ( 1.11 - 1.61 ) |
| Renal And Urinary Disorders | Renal Impairment | 109 | 10.75 ( 8.89 - 12.98 ) |
| Investigations | Urine Protein/Creatinine Ratio Increased | 94 | 1322.31 ( 1026.22 - 1703.82 ) |
| Musculoskeletal And Connective Tissue Disorders | Systemic Lupus Erythematosus | 90 | 11.14 ( 9.05 - 13.72 ) |
| Respiratory, Thoracic And Mediastinal Disorders | Cough | 79 | 1.52 ( 1.22 - 1.89 ) |
| Infections And Infestations | Urinary Tract Infection | 77 | 2.11 ( 1.68 - 2.64 ) |
| Blood And Lymphatic System Disorders | Anaemia | 69 | 2.57 ( 2.03 - 3.26 ) |
| Investigations | Laboratory Test Abnormal | 66 | 12.81 ( 10.05 - 16.33 ) |
| Infections And Infestations | Nasopharyngitis | 60 | 1.55 ( 1.2 - 1.99 ) |
| Metabolism And Nutrition Disorders | Decreased Appetite | 58 | 1.54 ( 1.19 - 2 ) |
| Gastrointestinal Disorders | Dyspepsia | 57 | 3.42 ( 2.63 - 4.44 ) |
| Nervous System Disorders | Migraine | 55 | 2.53 ( 1.94 - 3.29 ) |
| Gastrointestinal Disorders | Abdominal Pain | 52 | 1.33 ( 1.01 - 1.74 ) |
| Musculoskeletal And Connective Tissue Disorders | Joint Swelling | 49 | 1.87 ( 1.41 - 2.47 ) |
| Nervous System Disorders | Tremor | 45 | 1.68 ( 1.26 - 2.26 ) |
| Renal And Urinary Disorders | Acute Kidney Injury | 44 | 2.05 ( 1.52 - 2.76 ) |
| Infections And Infestations | Infection | 39 | 1.63 ( 1.19 - 2.23 ) |
| Gastrointestinal Disorders | Gastrointestinal Disorder | 39 | 2.61 ( 1.91 - 3.58 ) |
| Infections And Infestations | Influenza | 38 | 1.69 ( 1.23 - 2.32 ) |
| Metabolism And Nutrition Disorders | Dehydration | 33 | 1.69 ( 1.2 - 2.38 ) |
| Investigations | Heart Rate Increased | 31 | 1.82 ( 1.28 - 2.59 ) |
| Investigations | Protein Urine Present | 31 | 46.59 ( 32.64 - 66.52 ) |
| Infections And Infestations | Herpes Zoster | 30 | 2.64 ( 1.85 - 3.78 ) |
| Investigations | Haemoglobin Decreased | 28 | 1.98 ( 1.37 - 2.87 ) |
| Renal And Urinary Disorders | Renal Disorder | 25 | 3.67 ( 2.48 - 5.44 ) |
| Investigations | Blood Pressure Abnormal | 24 | 6.46 ( 4.32 - 9.64 ) |
| Metabolism And Nutrition Disorders | Fluid Retention | 21 | 2.2 ( 1.43 - 3.37 ) |
| Respiratory, Thoracic And Mediastinal Disorders | Epistaxis | 21 | 1.81 ( 1.18 - 2.78 ) |
| Investigations | Protein Total Increased | 19 | 53.05 ( 33.65 - 83.63 ) |
| Renal And Urinary Disorders | Pollakiuria | 15 | 2.45 ( 1.47 - 4.06 ) |
| Gastrointestinal Disorders | Flatulence | 15 | 1.67 ( 1.01 - 2.77 ) |
| Renal And Urinary Disorders | Urine Abnormality | 15 | 17.35 ( 10.43 - 28.84 ) |
| Metabolism And Nutrition Disorders | Hyperkalaemia | 15 | 4.47 ( 2.69 - 7.41 ) |
| Investigations | Blood Potassium Increased | 14 | 7.51 ( 4.44 - 12.7 ) |
| Metabolism And Nutrition Disorders | Hypervolaemia | 13 | 19.22 ( 11.13 - 33.19 ) |
| Infections And Infestations | Gastroenteritis Viral | 13 | 3.48 ( 2.02 - 5.99 ) |
| Vascular Disorders | Blood Pressure Fluctuation | 13 | 3.2 ( 1.86 - 5.52 ) |
| Gastrointestinal Disorders | Frequent Bowel Movements | 13 | 2.77 ( 1.61 - 4.78 ) |
| Investigations | Blood Urine Present | 13 | 4.96 ( 2.88 - 8.55 ) |
| Investigations | Blood Urea Increased | 13 | 9.03 ( 5.24 - 15.58 ) |
| Investigations | Blood Test Abnormal | 12 | 4.29 ( 2.43 - 7.55 ) |
| Skin And Subcutaneous Tissue Disorders | Hair Growth Abnormal | 11 | 7.37 ( 4.08 - 13.33 ) |
| Infections And Infestations | Kidney Infection | 11 | 2.42 ( 1.34 - 4.37 ) |
| Musculoskeletal And Connective Tissue Disorders | Joint Stiffness | 10 | 1.96 ( 1.06 - 3.65 ) |
| Investigations | Double Stranded Dna Antibody Positive | 10 | 197.41 ( 103.49 - 376.58 ) |
| Investigations | Blood Creatine Increased | 10 | 21.63 ( 11.6 - 40.33 ) |
| Reproductive System And Breast Disorders | Heavy Menstrual Bleeding | 10 | 5.63 ( 3.03 - 10.47 ) |
| Investigations | Glomerular Filtration Rate Abnormal | 10 | 57.37 ( 30.63 - 107.48 ) |
| Renal And Urinary Disorders | Lupus Nephritis | 9 | 29.07 ( 15.06 - 56.12 ) |
| Investigations | Complement Factor C3 Decreased | 9 | 268.76 ( 134.76 - 535.97 ) |
| Skin And Subcutaneous Tissue Disorders | Hypertrichosis | 8 | 18.5 ( 9.22 - 37.11 ) |
| Investigations | Blood Iron Decreased | 8 | 3.27 ( 1.64 - 6.55 ) |
| Metabolism And Nutrition Disorders | Gout | 8 | 4.37 ( 2.19 - 8.75 ) |
| Gastrointestinal Disorders | Gingival Swelling | 8 | 10.35 ( 5.17 - 20.73 ) |
| Investigations | Blood Albumin Decreased | 8 | 10.08 ( 5.03 - 20.18 ) |
| Investigations | Complement Factor C4 Decreased | 8 | 332.71 ( 158.57 - 698.09 ) |
| Gastrointestinal Disorders | Mouth Ulceration | 8 | 2.18 ( 1.09 - 4.35 ) |
| Investigations | Complement Factor Decreased | 8 | 372.64 ( 176.62 - 786.2 ) |
| Investigations | Blood Creatinine Abnormal | 8 | 17.09 ( 8.52 - 34.28 ) |
| Renal And Urinary Disorders | Renal Pain | 7 | 4.12 ( 1.96 - 8.65 ) |
| Gastrointestinal Disorders | Gingival Bleeding | 7 | 3.48 ( 1.66 - 7.3 ) |
| Reproductive System And Breast Disorders | Menstrual Disorder | 7 | 3.33 ( 1.58 - 6.98 ) |
| Immune System Disorders | Seasonal Allergy | 7 | 2.14 ( 1.02 - 4.5 ) |
| Investigations | Glomerular Filtration Rate Increased | 7 | 54.34 ( 25.68 - 114.99 ) |
| Investigations | Urine Albumin/Creatinine Ratio Increased | 7 | 153.79 ( 71.55 - 330.53 ) |
| Renal And Urinary Disorders | Haematuria | 7 | 2.21 ( 1.05 - 4.64 ) |
| Renal And Urinary Disorders | Urine Odour Abnormal | 6 | 5 ( 2.24 - 11.14 ) |
| Gastrointestinal Disorders | Haemorrhoids | 6 | 2.37 ( 1.06 - 5.27 ) |
| Metabolism And Nutrition Disorders | Iron Deficiency | 6 | 7.6 ( 3.41 - 16.94 ) |
| Investigations | Blood Sodium Decreased | 6 | 2.26 ( 1.01 - 5.03 ) |
| Vascular Disorders | Hypertensive Urgency | 6 | 81.7 ( 36.19 - 184.45 ) |
| Investigations | Urine Protein/Creatinine Ratio Abnormal | 6 | 481.76 ( 199.97 - 1160.64 ) |
| Nervous System Disorders | Brain Fog | 6 | 3.68 ( 1.65 - 8.2 ) |
| Renal And Urinary Disorders | Nocturia | 5 | 3.3 ( 1.37 - 7.93 ) |
| Investigations | Protein Total Decreased | 5 | 11.09 ( 4.6 - 26.7 ) |
| Renal And Urinary Disorders | Polyuria | 5 | 4.63 ( 1.93 - 11.14 ) |
| Nervous System Disorders | Posterior Reversible Encephalopathy Syndrome | 5 | 2.98 ( 1.24 - 7.17 ) |
| Investigations | Blood Creatinine Decreased | 5 | 10.25 ( 4.26 - 24.67 ) |
| Musculoskeletal And Connective Tissue Disorders | Flank Pain | 5 | 3.22 ( 1.34 - 7.74 ) |
| Investigations | Serology Abnormal | 5 | 1058.32 ( 367.64 - 3046.54 ) |
| Gastrointestinal Disorders | Breath Odour | 4 | 12.62 ( 4.72 - 33.72 ) |
| Investigations | Blood Potassium Abnormal | 4 | 9.17 ( 3.44 - 24.5 ) |
| Skin And Subcutaneous Tissue Disorders | Butterfly Rash | 4 | 15.37 ( 5.75 - 41.08 ) |
| Gastrointestinal Disorders | Gastrointestinal Tract Irritation | 4 | 20.11 ( 7.52 - 53.83 ) |
| Metabolism And Nutrition Disorders | Appetite Disorder | 4 | 3.51 ( 1.31 - 9.35 ) |
| Investigations | Blood Uric Acid Increased | 4 | 6.84 ( 2.56 - 18.25 ) |
| Investigations | Protein Total Abnormal | 4 | 18.7 ( 6.99 - 50.03 ) |
| Infections And Infestations | Pyuria | 4 | 31.89 ( 11.89 - 85.56 ) |
| Investigations | White Blood Cell Count Abnormal | 4 | 3.47 ( 1.3 - 9.25 ) |
| Vascular Disorders | Raynaud'S Phenomenon | 4 | 3.99 ( 1.5 - 10.65 ) |
| Gastrointestinal Disorders | Abnormal Faeces | 4 | 2.98 ( 1.12 - 7.95 ) |
| Investigations | Bacterial Test Positive | 4 | 10.43 ( 3.9 - 27.85 ) |
| Renal And Urinary Disorders | Nephritis | 4 | 9.79 ( 3.67 - 26.15 ) |
| Investigations | Renal Function Test Abnormal | 3 | 4.1 ( 1.32 - 12.71 ) |
| Blood And Lymphatic System Disorders | White Blood Cell Disorder | 3 | 6.36 ( 2.05 - 19.75 ) |
| Renal And Urinary Disorders | Nephrotic Syndrome | 3 | 4.64 ( 1.5 - 14.41 ) |
| Respiratory, Thoracic And Mediastinal Disorders | Acute Pulmonary Oedema | 3 | 4.68 ( 1.51 - 14.54 ) |
| Investigations | Protein Urine | 3 | 24.94 ( 7.99 - 77.81 ) |
| Eye Disorders | Eyelid Rash | 3 | 14.4 ( 4.63 - 44.81 ) |
| Respiratory, Thoracic And Mediastinal Disorders | Pleuritic Pain | 3 | 8.23 ( 2.65 - 25.56 ) |
| Vascular Disorders | Malignant Hypertension | 3 | 28.05 ( 8.98 - 87.57 ) |
| Infections And Infestations | Coccidioidomycosis | 3 | 19.67 ( 6.31 - 61.3 ) |
| Renal And Urinary Disorders | Microalbuminuria | 3 | 36.37 ( 11.63 - 113.79 ) |
| Renal And Urinary Disorders | Albuminuria | 3 | 67.8 ( 21.51 - 213.73 ) |
| Infections And Infestations | Streptobacillus Infection | 3 | 537.19 ( 153.05 - 1885.43 ) |
| Skin And Subcutaneous Tissue Disorders | Skin Depigmentation | 3 | 12.27 ( 3.95 - 38.17 ) |
| Musculoskeletal And Connective Tissue Disorders | Joint Lock | 3 | 5.59 ( 1.8 - 17.35 ) |
| Infections And Infestations | Escherichia Urinary Tract Infection | 3 | 3.61 ( 1.16 - 11.21 ) |

**Supplementary Table S5** Positive signals of voclosporin adverse events in the 18-64 age group.

| **SOC** | **PTs** | **Case numbers** | **ROR(95%Cl)** |
| --- | --- | --- | --- |
| Vascular Disorders | Hypertension | 221 | 12.53 ( 10.95 - 14.34 ) |
| Nervous System Disorders | Headache | 208 | 2.88 ( 2.51 - 3.31 ) |
| Gastrointestinal Disorders | Nausea | 165 | 2.04 ( 1.75 - 2.38 ) |
| Gastrointestinal Disorders | Diarrhoea | 148 | 2.54 ( 2.15 - 2.98 ) |
| Renal And Urinary Disorders | Proteinuria | 103 | 59.76 ( 49.07 - 72.77 ) |
| Gastrointestinal Disorders | Vomiting | 101 | 2.22 ( 1.82 - 2.7 ) |
| Gastrointestinal Disorders | Abdominal Pain Upper | 99 | 4.89 ( 4 - 5.96 ) |
| Infections And Infestations | Covid-19 | 90 | 3.57 ( 2.9 - 4.39 ) |
| Investigations | Blood Creatinine Increased | 88 | 16.92 ( 13.7 - 20.9 ) |
| Investigations | Glomerular Filtration Rate Decreased | 86 | 93.66 ( 75.43 - 116.31 ) |
| Investigations | Blood Pressure Increased | 85 | 6.09 ( 4.91 - 7.54 ) |
| Gastrointestinal Disorders | Abdominal Discomfort | 82 | 4.92 ( 3.96 - 6.12 ) |
| Investigations | Urine Protein/Creatinine Ratio Increased | 77 | 1446.27 ( 1095.32 - 1909.66 ) |
| Nervous System Disorders | Dizziness | 67 | 1.45 ( 1.14 - 1.85 ) |
| Musculoskeletal And Connective Tissue Disorders | Arthralgia | 62 | 1.34 ( 1.04 - 1.72 ) |
| Musculoskeletal And Connective Tissue Disorders | Systemic Lupus Erythematosus | 60 | 15.62 ( 12.1 - 20.16 ) |
| Renal And Urinary Disorders | Renal Impairment | 58 | 9.66 ( 7.46 - 12.52 ) |
| Respiratory, Thoracic And Mediastinal Disorders | Cough | 52 | 1.94 ( 1.47 - 2.55 ) |
| Skin And Subcutaneous Tissue Disorders | Alopecia | 47 | 2.05 ( 1.53 - 2.73 ) |
| Blood And Lymphatic System Disorders | Anaemia | 44 | 3.12 ( 2.32 - 4.2 ) |
| Renal And Urinary Disorders | Acute Kidney Injury | 41 | 2.55 ( 1.87 - 3.46 ) |
| Infections And Infestations | Urinary Tract Infection | 41 | 2.77 ( 2.03 - 3.76 ) |
| Metabolism And Nutrition Disorders | Decreased Appetite | 39 | 2.04 ( 1.49 - 2.8 ) |
| Gastrointestinal Disorders | Dyspepsia | 38 | 4.19 ( 3.04 - 5.76 ) |
| Nervous System Disorders | Tremor | 29 | 1.99 ( 1.38 - 2.87 ) |
| Musculoskeletal And Connective Tissue Disorders | Muscle Spasms | 28 | 1.55 ( 1.07 - 2.24 ) |
| Musculoskeletal And Connective Tissue Disorders | Joint Swelling | 27 | 1.9 ( 1.3 - 2.78 ) |
| Nervous System Disorders | Migraine | 26 | 2.08 ( 1.42 - 3.06 ) |
| Investigations | Laboratory Test Abnormal | 25 | 9.94 ( 6.7 - 14.73 ) |
| Infections And Infestations | Influenza | 23 | 1.78 ( 1.18 - 2.69 ) |
| Metabolism And Nutrition Disorders | Dehydration | 20 | 2.05 ( 1.32 - 3.18 ) |
| Investigations | Heart Rate Increased | 18 | 1.77 ( 1.11 - 2.81 ) |
| Infections And Infestations | Herpes Zoster | 17 | 2.93 ( 1.82 - 4.72 ) |
| Respiratory, Thoracic And Mediastinal Disorders | Epistaxis | 14 | 2.41 ( 1.42 - 4.07 ) |
| Renal And Urinary Disorders | Renal Disorder | 12 | 4.05 ( 2.3 - 7.14 ) |
| Investigations | Blood Urea Increased | 12 | 12.81 ( 7.26 - 22.6 ) |
| Investigations | Protein Urine Present | 11 | 20.71 ( 11.43 - 37.5 ) |
| Investigations | Blood Potassium Increased | 11 | 11.87 ( 6.56 - 21.47 ) |
| Metabolism And Nutrition Disorders | Hyperkalaemia | 11 | 4.85 ( 2.68 - 8.76 ) |
| Metabolism And Nutrition Disorders | Hypervolaemia | 10 | 25.41 ( 13.62 - 47.38 ) |
| Investigations | Blood Urine Present | 10 | 6.24 ( 3.35 - 11.6 ) |
| Vascular Disorders | Blood Pressure Fluctuation | 10 | 4.22 ( 2.27 - 7.85 ) |
| Renal And Urinary Disorders | Urine Abnormality | 8 | 14.8 ( 7.38 - 29.67 ) |
| Investigations | Double Stranded Dna Antibody Positive | 8 | 207.53 ( 101.03 - 426.29 ) |
| Renal And Urinary Disorders | Pollakiuria | 8 | 2.44 ( 1.22 - 4.88 ) |
| Investigations | Blood Creatine Increased | 8 | 25.39 ( 12.65 - 50.95 ) |
| Metabolism And Nutrition Disorders | Gout | 7 | 5.92 ( 2.82 - 12.44 ) |
| Renal And Urinary Disorders | Lupus Nephritis | 6 | 25.32 ( 11.33 - 56.59 ) |
| Investigations | Blood Pressure Abnormal | 6 | 3.97 ( 1.78 - 8.85 ) |
| Investigations | Blood Albumin Decreased | 6 | 9.92 ( 4.45 - 22.11 ) |
| Infections And Infestations | Laryngitis | 6 | 5.63 ( 2.53 - 12.56 ) |
| Investigations | Glomerular Filtration Rate Abnormal | 6 | 40.99 ( 18.29 - 91.83 ) |
| Infections And Infestations | Gastroenteritis Viral | 6 | 2.92 ( 1.31 - 6.51 ) |
| Renal And Urinary Disorders | Haematuria | 6 | 2.54 ( 1.14 - 5.66 ) |
| Investigations | Urine Albumin/Creatinine Ratio Increased | 6 | 144.38 ( 63.47 - 328.44 ) |
| Skin And Subcutaneous Tissue Disorders | Hair Growth Abnormal | 5 | 9.63 ( 4 - 23.19 ) |
| Skin And Subcutaneous Tissue Disorders | Hypertrichosis | 5 | 22.59 ( 9.37 - 54.5 ) |
| Gastrointestinal Disorders | Haemorrhoids | 5 | 3.32 ( 1.38 - 7.98 ) |
| Investigations | Complement Factor C3 Decreased | 5 | 178.04 ( 71.98 - 440.38 ) |
| Metabolism And Nutrition Disorders | Feeding Disorder | 5 | 2.79 ( 1.16 - 6.72 ) |
| Gastrointestinal Disorders | Gingival Swelling | 5 | 12.42 ( 5.16 - 29.91 ) |
| Gastrointestinal Disorders | Gingival Bleeding | 5 | 4.08 ( 1.7 - 9.81 ) |
| Metabolism And Nutrition Disorders | Iron Deficiency | 5 | 11.52 ( 4.78 - 27.74 ) |
| Nervous System Disorders | Posterior Reversible Encephalopathy Syndrome | 5 | 4.44 ( 1.85 - 10.68 ) |
| Vascular Disorders | Hypertensive Urgency | 5 | 105.14 ( 43 - 257.06 ) |
| Investigations | Urine Protein/Creatinine Ratio Abnormal | 5 | 460.44 ( 178.17 - 1189.92 ) |
| Renal And Urinary Disorders | Polyuria | 4 | 4.94 ( 1.85 - 13.18 ) |
| Investigations | Renal Function Test Abnormal | 4 | 12.26 ( 4.59 - 32.76 ) |
| Reproductive System And Breast Disorders | Menstrual Disorder | 4 | 3.54 ( 1.33 - 9.43 ) |
| Reproductive System And Breast Disorders | Heavy Menstrual Bleeding | 4 | 4.27 ( 1.6 - 11.38 ) |
| Gastrointestinal Disorders | Faeces Soft | 4 | 4.75 ( 1.78 - 12.66 ) |
| Investigations | Complement Factor Decreased | 4 | 368.29 ( 129.43 - 1047.95 ) |
| Investigations | Glomerular Filtration Rate Increased | 4 | 37.47 ( 13.96 - 100.57 ) |
| Investigations | White Blood Cell Count Abnormal | 4 | 8.23 ( 3.08 - 21.96 ) |
| Investigations | Bacterial Test Positive | 4 | 14.61 ( 5.47 - 39.05 ) |
| Gastrointestinal Disorders | Breath Odour | 3 | 14.2 ( 4.56 - 44.17 ) |
| Blood And Lymphatic System Disorders | Increased Tendency To Bruise | 3 | 3.97 ( 1.28 - 12.31 ) |
| Investigations | Protein Total Increased | 3 | 11.52 ( 3.71 - 35.83 ) |
| Investigations | Complement Factor C4 Decreased | 3 | 160.18 ( 49.95 - 513.7 ) |
| Investigations | Blood Uric Acid Increased | 3 | 6.67 ( 2.15 - 20.71 ) |
| Skin And Subcutaneous Tissue Disorders | Butterfly Rash | 3 | 18.41 ( 5.91 - 57.32 ) |
| Gastrointestinal Disorders | Gastrointestinal Tract Irritation | 3 | 26.17 ( 8.39 - 81.63 ) |
| Gastrointestinal Disorders | Food Poisoning | 3 | 4.96 ( 1.6 - 15.4 ) |
| Investigations | Protein Urine | 3 | 32.56 ( 10.42 - 101.67 ) |
| Renal And Urinary Disorders | Nephrotic Syndrome | 3 | 5.66 ( 1.82 - 17.57 ) |
| Respiratory, Thoracic And Mediastinal Disorders | Acute Pulmonary Oedema | 3 | 7.29 ( 2.35 - 22.64 ) |
| Infections And Infestations | Pyuria | 3 | 41.71 ( 13.33 - 130.52 ) |
| Vascular Disorders | Malignant Hypertension | 3 | 40.65 ( 13 - 127.18 ) |
| Investigations | Blood Creatinine Abnormal | 3 | 11.51 ( 3.7 - 35.78 ) |
| Vascular Disorders | Raynaud'S Phenomenon | 3 | 5.57 ( 1.79 - 17.28 ) |
| Nervous System Disorders | Brain Fog | 3 | 3.6 ( 1.16 - 11.18 ) |
| Investigations | Blood Creatinine Decreased | 3 | 8.79 ( 2.83 - 27.32 ) |
| Renal And Urinary Disorders | Microalbuminuria | 3 | 49.44 ( 15.77 - 154.94 ) |
| Eye Disorders | Swelling Of Eyelid | 3 | 4.7 ( 1.51 - 14.6 ) |
| Infections And Infestations | Streptobacillus Infection | 3 | 728.08 ( 203.06 - 2610.51 ) |
| Investigations | Serology Abnormal | 3 | 2002.22 ( 448.01 - 8948.25 ) |

**Supplementary Table S6** Positive signals of voclosporin adverse events in the age group 65 and above.

| **SOC** | **PTs** | **Case numbers** | **ROR(95%Cl)** |
| --- | --- | --- | --- |
| Vascular Disorders | Hypertension | 16 | 11.73 ( 7.11 - 19.38 ) |
| Gastrointestinal Disorders | Diarrhoea | 16 | 3.36 ( 2.04 - 5.55 ) |
| Nervous System Disorders | Headache | 8 | 2.98 ( 1.48 - 6.01 ) |
| Gastrointestinal Disorders | Abdominal Pain Upper | 7 | 6.13 ( 2.9 - 12.95 ) |
| Blood And Lymphatic System Disorders | Anaemia | 7 | 3.94 ( 1.86 - 8.32 ) |
| Gastrointestinal Disorders | Abdominal Discomfort | 7 | 7.18 ( 3.4 - 15.18 ) |
| Gastrointestinal Disorders | Vomiting | 7 | 2.94 ( 1.39 - 6.21 ) |
| Renal And Urinary Disorders | Proteinuria | 6 | 55.62 ( 24.79 - 124.76 ) |
| Investigations | Blood Creatinine Increased | 5 | 10.06 ( 4.16 - 24.32 ) |
| Investigations | Glomerular Filtration Rate Decreased | 5 | 45.73 ( 18.9 - 110.66 ) |
| Infections And Infestations | Covid-19 | 4 | 2.71 ( 1.01 - 7.25 ) |
| Musculoskeletal And Connective Tissue Disorders | Joint Swelling | 4 | 5.35 ( 2 - 14.34 ) |
| Musculoskeletal And Connective Tissue Disorders | Arthritis | 3 | 5.58 ( 1.79 - 17.4 ) |
| Metabolism And Nutrition Disorders | Fluid Retention | 3 | 7.08 ( 2.27 - 22.05 ) |
| Nervous System Disorders | Memory Impairment | 3 | 3.6 ( 1.15 - 11.21 ) |
| Metabolism And Nutrition Disorders | Hyperkalaemia | 3 | 7.47 ( 2.4 - 23.29 ) |
| Investigations | White Blood Cell Count Decreased | 3 | 3.37 ( 1.08 - 10.49 ) |
| Gastrointestinal Disorders | Dry Mouth | 3 | 5.89 ( 1.89 - 18.35 ) |

**Supplementary Table S7** Analysis results of the onset times of adverse events in investigations.

| **SOC** | **Cases** | **Time to onset** | | |
| --- | --- | --- | --- | --- |
|  |  | **Median** | **IQR** | **Min-Max** |
|  |  |  |  |  |
| Blood creatinine increased | 68 | 143 | 62.25-329 | 1-702 |
| Glomerular filtration rate decreased | 61 | 222 | 59.5-436 | 2-892 |
| Urine protein/creatinine ratio increased | 59 | 251 | 109-550 | 2-892 |
| Blood pressure increased | 53 | 28 | 6-156.5 | 1-549 |
